# Theoretical study on the generation of Criegee intermediates from the ozonolysis of trifluoropropene (CF_3_CH=CH_2_)

**DOI:** 10.1039/d5ra07265d

**Published:** 2025-12-15

**Authors:** Yunju Zhang, Meilian Zhao, Cen Yao, Zhiguo Wang, Yuxi Sun

**Affiliations:** a Key Laboratory of Photoinduced Functional Materials, Key Laboratory of Inorganic Materials Preparation and Synthesis, Mianyang Normal University Mianyang 621000 PR China zhangyj010@nenu.edu.cn +86 816 2200819 +86 816 2200064; b School of Public Health, Chengdu University of Traditional Chinese Medicine ChengDu PR China; c School-enterprise Joint Technology Innovation Laboratory of Novel Molecular Functional Materials of Jilin Province, Institute of Chemical and Industrial Bioengineering, Jilin Engineering Normal University Changchun 130052 China yaoc773@nenu.edu.cn

## Abstract

The O_3_-initiated degradation mechanisms of trifluoropropene (CF_3_CH=CH_2_) were studied using density functional theory (DFT). Three types of mechanisms were observed for the title reaction, namely, addition/elimination, H-abstraction and substitution. The computations showed that O_3_ with a C=C bond undergoes a 1,3-cycloaddition reaction to generate a primary ozone intermediate (POZ) with a relatively low free energy barrier, which then dissociates to generate an aldehyde group and carbonyl oxide, known as Criegee intermediates (CIs). Detailed analysis was conducted on the subsequent reactions of CIs. It is found that when a new type of CI-containing halogenated alkyl groups reacts with NO, NO_2_, CH_2_O, SO_2_, H_2_O and O_2_, its reaction pathway is singularly analogous to that of the general CI. The degradation total rate coefficient and the estimated lifetime are in accordance with the experimental results. The current calculation results are of great significance for the atmospheric chemistry of the ozone oxidation of unsaturated halogenated compounds.

## Abstract 2

The O_3_-initiated degradation mechanisms of trifluoropropene (CF_3_CH=CH_2_) were studied using density functional theory (DFT).

## Graphical abstract

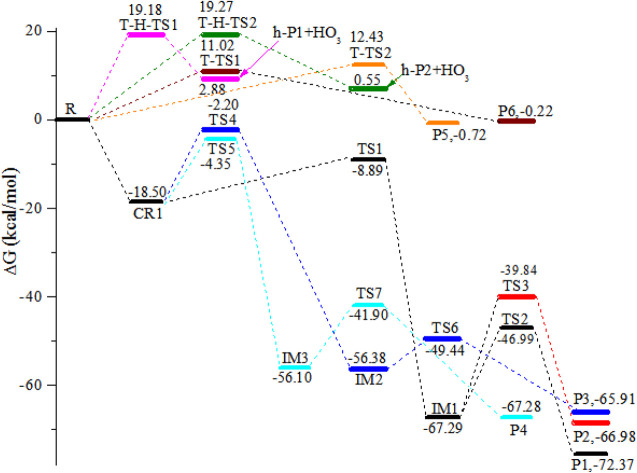

## 1. Introduction

The international community has realized that chlorofluorocarbons (CFCs) can have adverse effects on stratospheric ozone and induce greenhouse effects, urging researchers to work towards finding environmentally friendly alternatives to these compounds.^1,2^ Due to the absence of chlorine atoms in hydrofluorocarbons (HFCs), they are not conducive to the establishment of a good chlorine-based catalytic ozone-depletion cycle.^3^ Therefore, saturated hydrofluorocarbons (HFCs) have proven to be the extensively employed alternatives to CFCs. Unsaturated HFCs are latent alternatives to CFCs and saturated HFCs. The oxidation of the unsaturated HFCs could be induced by reactions with OH,^4–6^ O_3_ (ref. 7–10) and NO_3_ (ref. 9, 11 and 12) radicals in the atmosphere. Ozone decomposition in the atmosphere is one of the common oxidation channels for unsaturated hydrocarbons, which plays a significant role in both the city and the countryside.^13^ The reaction between O_3_ and unsaturated HFCs has been proven to involve the addition of double-bond systems to generate an ozonide, which rapidly dissociates to generate carbonyl oxides (Criegee intermediates (CI)) and aldehyde compounds. The reactions involving propylene and halogenated propylenes have recently attracted substantial experimental attention.^14–22^ Only one research group has investigated the kinetics of the reaction of CF_3_CH=CH_2_ with O_3_. In 2005, Sulbaek Andersen *et al.*^17^ investigated the kinetics and mechanism of the reaction of CF_3_CH=CH_2_ with O_3_ in an N_2_ atmosphere at 296 K by employing long-path-length FT-IR-smog chamber techniques. The measured rate constant of CF_3_CH=CH_2_ with O_3_ at 1 atm and 298 K is *k*_CF_3_CH=CH_2_+O_3__ = (3.50 ± 0.30) × 10^−19^ cm^3^ per molecule per s. The atmospheric lifetime of CF_3_CH=CH_2_ is approximately 70 days due to the reaction with O_3_. Unfortunately, thus far, the reaction between O_3_ and CF_3_CH=CH_2_ has not been well established theoretically. In the present work, to assess the oxidation reaction of CF_3_CH=CH_2_ induced by ozone, the reaction mechanism and the secondary pathways in the presence of NO, NO_2_, CH_2_O, H_2_O, SO_2_ and O_2_ are investigated by employing theoretical methods, which have been extensively employed in the environmental area.^23–31^ The rate constants of primary reaction pathways are predicted using the Rice–Ramsperger–Kassel–Marcus (RRKM) theory^32^ to forecast the atmospheric lifetime.

Up to date, very little is known about the final products and mechanisms of O_3_ with trifluoropropene (CF_3_CH=CH_2_). Moreover, the data on the Criegee intermediates with an aldehyde group and a trifluoromethyl group are scarce. Hence, we investigate the ozonolysis of trifluoropropene using quantum chemical methods. This work provides a comprehensive study on the reaction between trifluoropropene and ozone, which contributes to the literature on the mechanism of the primary step of ozone decomposition and the further reaction of CI. The research data in this work can help us evaluate the impact of these compounds on the atmosphere.

## 2 Computational methods

All the electronic structure computations for the CF_3_CH=CH_2_ + O_3_ reaction were performed using the Gaussian 09 program.^33^ The geometries of all stable points (including reactants, complexes, transition states, intermediates, and products) were optimized using the M06-2X^34^ method in combination with the 6-311++g(d,p) basis set,^35,36^ and the vibrational frequency analysis computations were performed at the same level. All the minima have real frequencies, while the transition states have only one imaginary frequency. Intrinsic reaction coordinate (IRC)^37,38^ computations were carried out to determine the transition states connecting the corresponding reactants and products. The CCSD(T)^39^//cc-pVTZ method was employed for all the species involved in the CF_3_CH=CH_2_ + O_3_ reaction to obtain accurate single-point energies.

## 3 Results and discussion

**Fig. 1:**
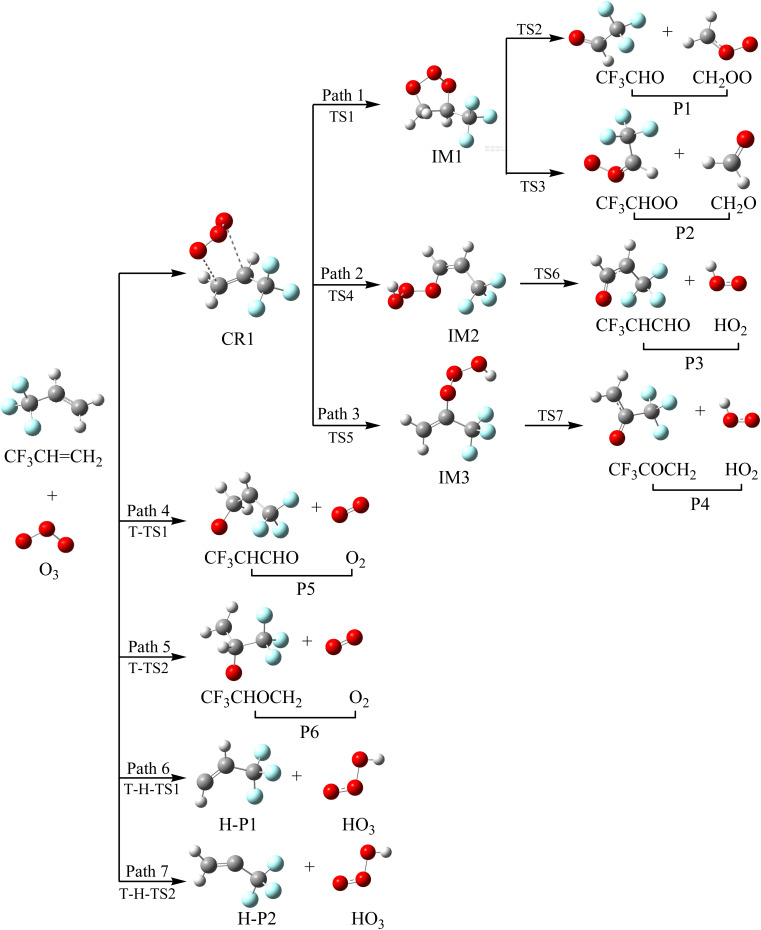
Optimized geometries and reaction schematic of the trifluoropropene (CF_3_CH=CH_2_) with ozone reaction.

**Fig. 2:**
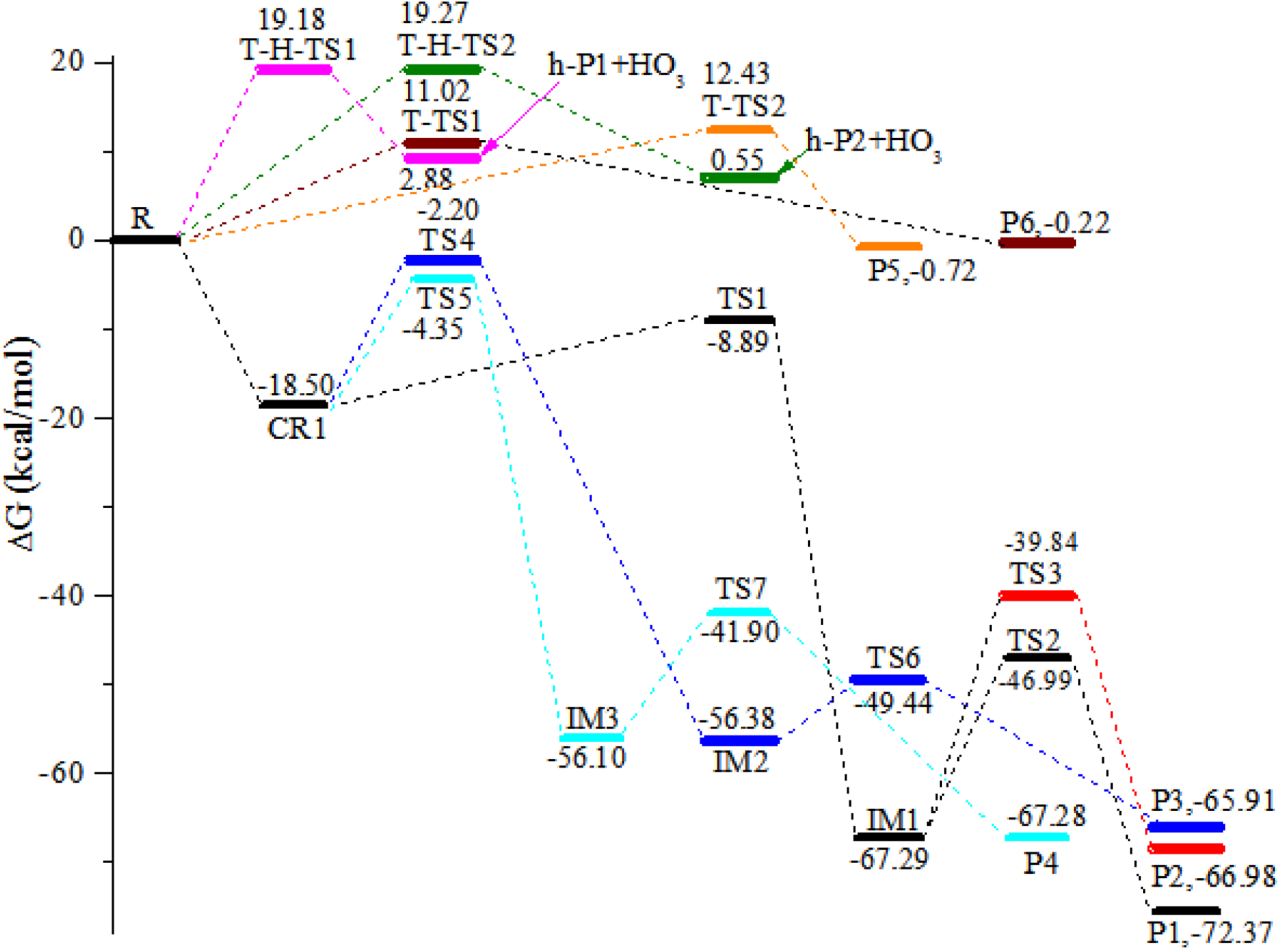
Potential energy surface for the reaction of trifluoropropene (CF_3_CH=CH_2_) with ozone calculated at the CCSD(T)/cc-pVTZ//M06-2X/6-311++g(d,p) level of theory.

Taking the reaction between trifluoropropene and ozone as an example, we describe the generation mechanism of POZ (primary ozone intermediate) and CIs (Criegee intermediates). All the optimized structures of the reactants, products and intermediates and the reaction schematics for the trifluoropropene with ozone reaction are presented in Fig. 1. All the transition states involved in the CF_3_CH=CH_2_ + O_3_ reaction at the M06-2X/6-311++g(d,p) level of theory are presented in Fig. S1. As can be seen from Fig. 1, the reaction of CF_3_CH=CH_2_ with O_3_ has a total of seven pathways, which mainly include three reaction mechanisms, namely addition/elimination, substitution and hydrogen abstraction. The potential energy surface (PES) of the CF_3_CH=CH_2_ + O_3_ reaction is presented in Fig. 2, and R in Fig. 2 represents the CF_3_CH=CH_2_ + O_3_. Table 1 summarizes the relative energies, reaction enthalpies and Gibbs free energy of all the species involved in the trifluoropropene with ozone reaction.

**Table 1:** Relative energies, reaction enthalpies and Gibbs free energy of all the species involved in the trifluoropropene with ozone reaction at 298 K (in kcal mol^−1^)

Species	Δ*E*	Δ*H*	Δ*G*
CF_3_CH=CH_2_ + O_3_	0.00	0.00	0.00
CR1	−28.47	−28.10	−18.50
IM1	−80.50	−81.84	−67.29
IM2	−67.81	−68.25	−56.38
IM3	−68.34	−68.91	−56.10
TS1	−21.35	−22.17	−8.89
TS2	−59.96	−61.07	−46.99
TS3	−52.75	−53.80	−39.84
TS4	−14.69	−15.58	−2.20
TS5	−16.64	−17.32	−4.35
TS6	−62.09	−63.02	−49.44
TS7	−53.09	−53.38	−41.90
T-TS1	0.75	0.61	11.02
T-TS2	1.83	1.67	12.43
T-H-TS1	9.24	9.32	19.18
T-H-TS2	9.42	9.56	19.27
P1	−75.46	−72.52	−72.37
P2	−68.37	−68.10	−66.98
P3	−65.81	−65.29	−65.91
P4	−68.08	−68.07	−67.28
P5	−2.41	−1.90	−0.72
P6	−1.28	−0.74	−0.22
H-P1 + HO_3_	9.30	4.30	2.88
H-P2 + HO_3_	7.05	2.12	0.55

### 3.1 Reaction mechanism of addition/elimination

As seen from Fig. 1, the two terminal oxygen atoms of the ozone molecule can simultaneously interact with the C=C bond in CF_3_CH=CH_2_ to generate the pre-complex (CR1). The relative energy of CR1 is −28.47 kcal mol^−1^, and the Δ*G* is −18.50 kcal mol^−1^. Subsequently, CR1 surmounts a free energy barrier of 9.61 kcal mol^−1^ through a transition state (TS1) to generate the primary ozone intermediate, a five-membered ring structure (IM1). The lengths of the two generated C–O bonds in TS1 are 2.20 and 2.22 Å. The two generated C–O bonds in IM1 have equal bond lengths of 1.41 Å. The generation of the intermediate (IM1) released a large amount of heat (81.84 kcal mol^−1^). The energy-rich intermediate (IM1) (80.50 kcal mol^−1^) could overcome the free energy barriers of 20.30 and 27.45 kcal mol^−1^ to generate P1 (CF_3_CHO + CH_2_OO (CI1)) and P2 (CF_3_CHOO (CI2) + CH_2_O), respectively. The transition states corresponding to the above two direct decomposition processes are TS2 and TS3, respectively. The lengths of the broken C–C and O–O bonds in TS2 and TS3 are 1.952 and 2.032 Å and 1.971 and 2.079 Å, respectively. The energies of P1 and P2 are lower than those of the reactants by 75.46 and 68.37 kcal mol^−1^, respectively. At the same time, 72.52 and 68.10 kcal mol^−1^ of heat are released to generate P1 and P2, respectively.

Starting from CR1, a terminal oxygen atom in the O_3_ molecule is added to the two carbon atoms of the C=C double bond in CF_3_CH=CH_2_. Thereafter, the H atoms in the –CH_2_ and –CH– groups shift to the other terminal oxygen atom in the O_3_ molecule *via* transition states TS4 and TS5, respectively. The free energy barriers of CR1 → TS4 → IM2 and CR1 → TS5 → IM3 are 16.30 and 14.15 kcal mol^−1^, respectively. The energies of IM2 and IM3 are −67.81 and −68.34 kcal mol^−1^, respectively. IM2 and IM3 can generate the final products (P3: CF_3_CHCHO + HO_2_ and P4: CF_3_COCH_2_ + HO_2_) through TS6 and TS7, respectively, by breaking the O–O bond. The free energy barriers for IM2 → TS6 → P3 and IM2 → TS7 → P4 are 6.94 and 14.20 kcal mol^−1^, respectively. However, because of the relatively high energy barriers for the addition process, the contribution of these two addition/elimination channels to the reaction may be relatively small, which will be proven in Section 3.5 (see Fig. 11).

### 3.2 Reaction mechanism of substitution

One of the end-group oxygen atoms in the O_3_ molecule is also added to the carbon atoms of the C=C double bond in CF_3_CH=CH_2_, which is associated with the breaking of the O–O bond to generate P5 (CF_3_CHOCH_2_ + O_2_(^1^Δ)) and P6 (CF_3_CHCH_2_O + O_2_(^1^Δ)) *via* triplet transition states T-TS1 and T-TS2, respectively. The corresponding free energy barriers for R → T-TS1 → P5 and R → T-TS2 → P6 are 11.02 and 12.43 kcal mol^−1^, respectively; hence, the importance of this channel for the reaction may be at higher temperature.

### 3.3 Reaction mechanism of hydrogen abstraction

It is worth mentioning that we did not find a transition state for hydrogen extraction on the singlet PES. While on the triplet PES, it was found that ozone can extract the hydrogen atoms on the –CH_2_ and –CH groups in the CF_3_CH=CH_2_ molecule to generate products H-P1 + HO_3_ and H-P2 + HO_3_*via* transition states T-H-TS1 and T-H-TS2, respectively. The two hydrogen abstraction reactions have free energy barriers of 19.18 and 19.27 kcal mol^−1^, respectively. The lengths of the broken C–H bonds in T-H-TS1 and T-H-TS2 are 1.281 and 1.289 Å, respectively, and the lengths of the generated O–H bonds are 1.212 and 1.202 Å, respectively. The energies of H-P1 + HO_3_ and H-P2 + HO_3_ are 9.30 and 7.05 kcal mol^−1^, respectively. Owing to the relatively high free energy barriers and unstable products, the contributions of the hydrogen extraction reaction channels on the triplet PES to the reaction are negligible.

### 3.4 Further reactions of the Criegee intermediates

**Fig. 3:**
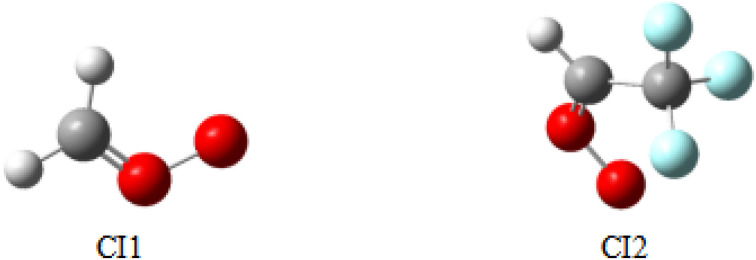
Optimized structure of the Criegee intermediates.

**Fig. 4:**
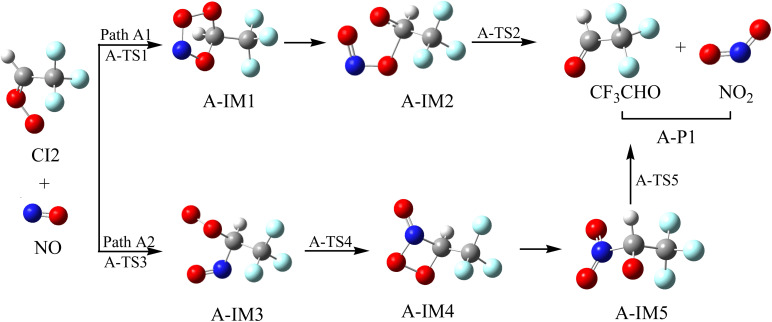
Optimized geometrical configurations in the reaction of CI2 with NO.

**Fig. 5:**
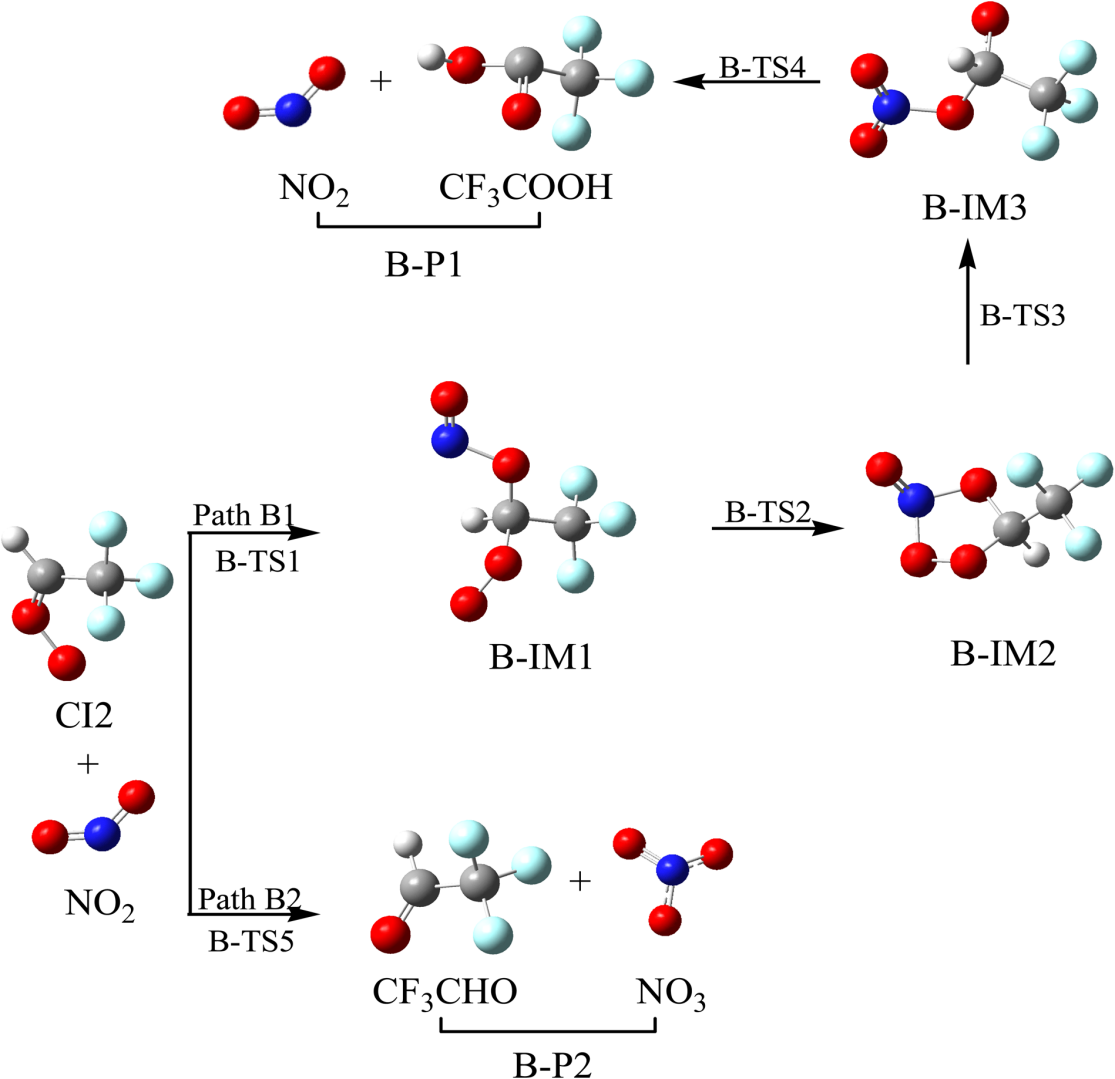
Optimized geometrical configuration and the reaction schematic of the CI2 with NO_2_ reaction.

**Fig. 6:**
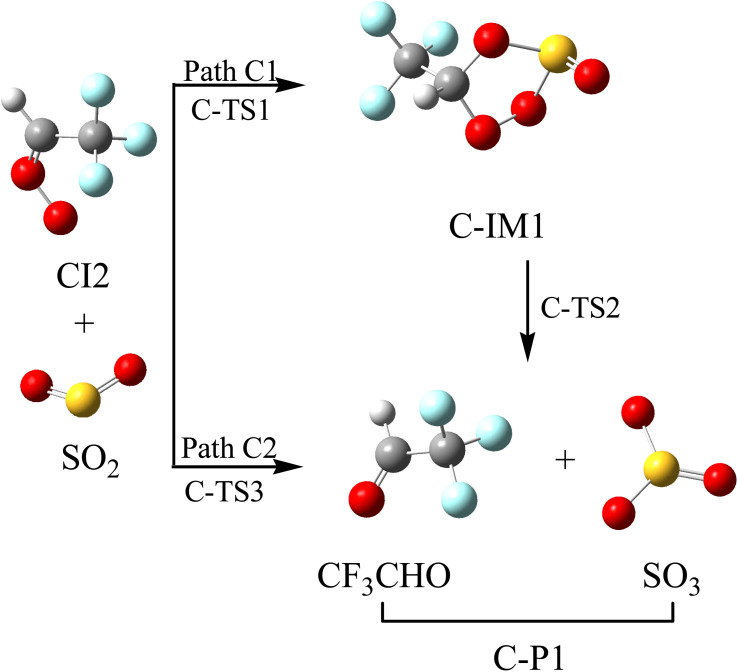
Optimized geometrical configuration and the reaction schematic of the CI2 with SO_2_ reaction.

**Fig. 7:**
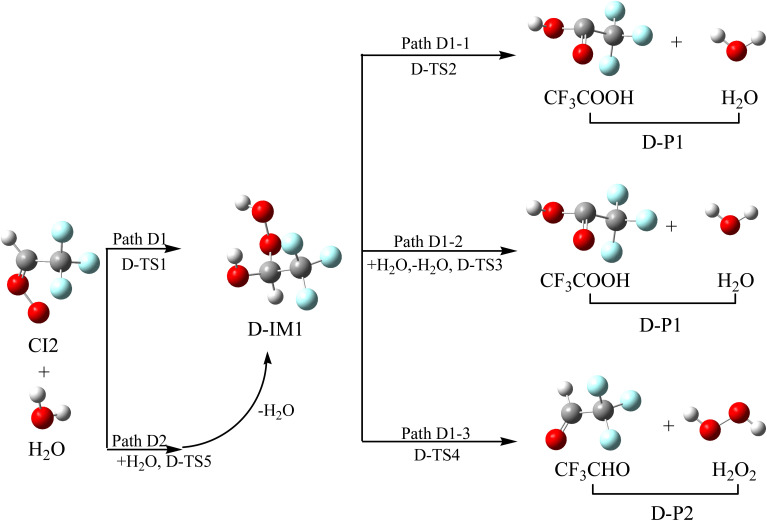
Optimized geometrical configuration and the reaction schematic of the CI2 with H_2_O reaction.

**Fig. 8:**
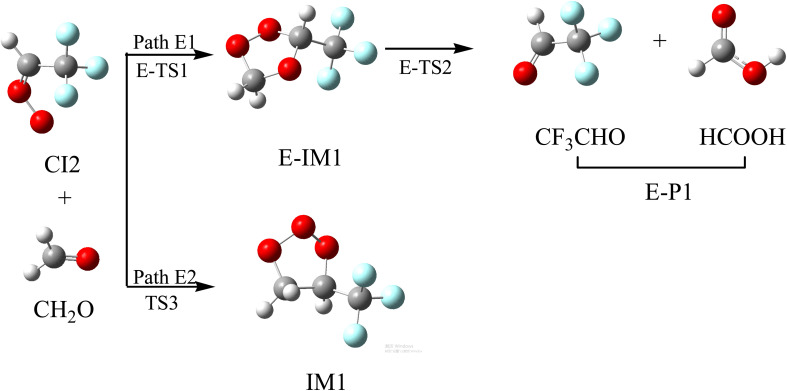
Optimized geometrical configuration and the reaction schematic of the CI2 with CH_2_O reaction.

**Fig. 9:**
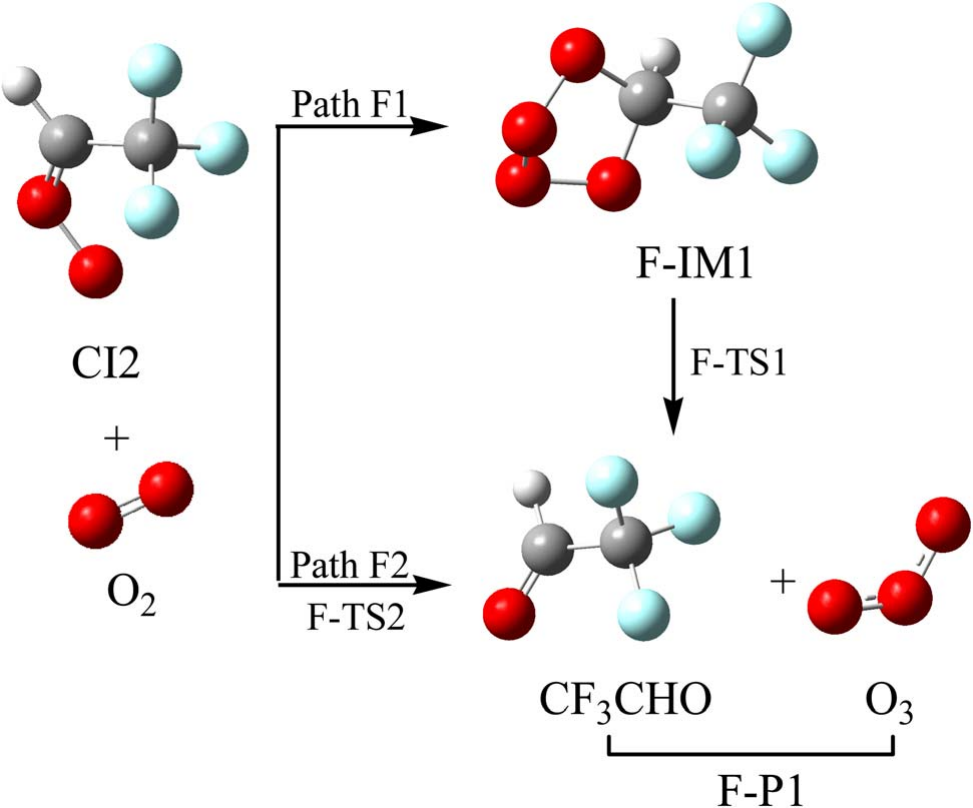
Optimized geometric configurations and the reaction schematic of the CI2 with O_2_ reaction.

The different Criegee intermediates resulting from the ozonation of trifluoropropene are named CI1 (CH_2_OO) and CI2 (CF_3_CHOO). CI1 is commonly found in the reaction of O_3_ with olefins. The optimized structures of CI1 and CI2 are shown in Fig. 3. The calculated bond lengths of C–O and O–O in CI1 (CH_2_OO) are consistent with the results observed in the previous investigation.^40^ CI2 is a new class of CIs with aldehyde groups, and it may have different properties. Therefore, this section presents the reaction mechanisms of Cl2 with NO, NO_2_, SO_2_, CH_2_O, H_2_O and O_2_. In CI2, the carbon atom in the –CH– group is the electrophilic reagent. Therefore, the nucleophilic atoms of NO, NO_2_, SO_2_, CH_2_O, H_2_O and O_2_ tend to attack the C atom, and the corresponding reaction proceeds, and the PESs are presented in Fig. 4–9 and S2–S7, respectively. All the transition states involved in the reactions of CF_3_CHOO with NO, NO_2_, CH_2_O, H_2_O, SO_2_ and O_2_ at the M06-2X/6-311++g(d,p) level of theory are presented in Fig. S8. These results indicate that the final products from the CI reactions are common atmospheric substances, such as saturated trifluoroacetaldehyde, trifluoroacetic acid and formic acid, as the content of SOA. The present study enriches the literature on the types of CIs and plays a significant role in controlling atmospheric chemical pollution, which will be discussed in detail in the following sections.

#### 3.4.1 Reaction mechanism of CI2 with NO

The reaction schematics, including the optimized geometries and the PES of the Criegee intermediate (CF_3_CHOO(CI2)) with NO reaction, are presented in Fig. 4 and S2, respectively. There are two different addition/isomerization/elimination reaction channels (Path A1 and Path A2) for the reaction of CI2 with NO. For Path A1, the N and O atoms in NO can be simultaneously added to the terminal-O and C atoms of the –CH group in CI2 through A-TS1 to generate a five-membered ring intermediate, A-IM1. This process requires the crossing of a free energy barrier of 18.89 kcal mol^−1^. A-IM1, with an internal energy of 18.30 kcal mol^−1^, could directly break the O–O bond to generate A-IM2. It is worth noting that this decomposition process is a barrier-free process, and A-IM2 directly breaks the C–O bond to generate the final product (A-P1 (CF_3_CHO + NO_2_)) through A-TS2, surmounting a free energy barrier of 13.95 kcal mol^−1^. For Path A2, the N atom in NO could also be added to the C atom in the –CH group of CI2, crossing a free energy barrier of 11.76 kcal mol^−1^*via* A-TS3 to generate A-IM3. Subsequently, A-IM3 passes through A-TS4 to generate a tetradentate cyclic intermediate (A-IM4) by interacting the N atom with the end-group O atom of –COO, overcoming a free energy barrier of 14.34 kcal mol^−1^. A-IM4 can directly break both the O–O and C–N bonds to generate A-IM5. This direct decomposition process is also a barrier-free process. A-IM5 can eliminate an NO_2_ to generate A-P1 (CF_3_CHO + NO_2_) through A-TS5 after overcoming a negative free energy barrier of 72.03 kcal mol^−1^, indicating that NO_2_ loss is easy.

#### 3.4.2 Reaction mechanism of CI2 with NO_2_

It was found that the CI2 + NO_2_ reaction has two pathways, *i.e.*, the addition/isomerization/elimination pathway (Path B1) and the substitution pathway (Path B2). The reaction schematics, including the optimized geometries and the PES of this reaction, are presented in Fig. 5 and S3, respectively. As for Path B1, one of the O atoms in NO_2_ could add to the C atom in the –CH group in CI2 to generate B-IM1 *via* B-TS1. This addition process requires overcoming a free energy barrier of 17.19 kcal mol^−1^. The length of the C–O bond in B-TS1 is 2.088 Å. The N atom in B-IM1 could interact with the terminal oxygen atom in –COO through B-TS2 to generate a five-membered ring intermediate (B-IM2), surmounting a free energy barrier of 29.82 kcal mol^−1^. Subsequently, the five-membered ring intermediate (B-IM2) could be opened by breaking the O–O bond to generate B-IM3 through B-TS3, and then, it undergoes the synergistic reaction of 1,3-H migration and O–N bond breaking to generate B-P1 (CF_3_COOH + NO_2_) through B-TS4. The free energy barrier height of B-IM2 → B-TS3 → B-IM3 and B-IM3 → B-TS4 → B-P1 are 2.29 and 31.70 kcal mol^−1^, respectively. The generation of B-P1 is exothermic, with an internal energy of 117.88 kcal mol^−1^. For Path B2, the N atom in the NO_2_ radical can attack the terminal-O atom in CI2, accompanied by breaking the O–O bond to generate B-P2 (CF_3_CHO + NO_3_) through B-TS5. The free energy barrier of CI2 + NO_2_ → B-TS5 → B-P2 is 23.74 kcal mol^−1^, which is 6.55 kcal mol^−1^ higher than that of CI2 + NO_2_ → B-TS1 → B-IM1. Thus, Path B1 is superior to Path B2. These reactions can supply some resources for NO_3_ radicals and CF_3_COOH radicals.

#### 3.4.3 Reaction mechanism of CI2 with SO_2_

CI2 is able to oxidize SO_2_ to generate SO_3_, which then generates a sulfuric acid aerosol.^41–45^ The reaction schematics, including the optimized geometries and the PES of this reaction, are presented in Fig. 6 and S4, respectively. The cycloaddition/elimination (Path C1) and substitution (Path C2) reaction mechanisms have been found for the CI2 with SO_2_ reaction. For Path C1, the S atom and one of the oxygen atoms in SO_2_ could be simultaneously added to the terminal oxygen atom and the C atom in the –CH group in CI2 to generate a five-membered cycloaddition intermediate (C-IM1) through transition state C-TS1, surmounting a free energy barrier of 3.38 kcal mol^−1^. The chemically activated adduct (C-IM1) (41.86 kcal mol^−1^) could directly dissociate to the final product (C-P1: CF_3_CHO + SO_3_) through C-TS2, and it involves simultaneously breaking the C–O and O–O bonds. The free energy barrier for the process of C-IM1 → C-TS2 → C-P1 is 16.24 kcal mol^−1^. Similar to the reaction between CI2 and NO_2_, the S atom in SO_2_ attacks the terminal-O atom of CI2, kicking off the CF_3_CHO to give out SO_3_ through C-TS3. The free energy barrier of C-IM1 → C-TS3 → C-P1 is 20.76 kcal mol^−1^, indicating that the generation of SO_3_ through Path C1 is relatively easier than that through Path C2. Furthermore, these pathways will contribute to the generation of SOA.

#### 3.4.4 Reaction mechanism of CI2 with H_2_O

The investigation on the reaction between CI and H_2_O primarily concentrates on the reaction between the simplest Criegee intermediate (CH_2_OO) and water vapor, and the comprehensive theoretical investigation shows that the water dimer pathway dominates and water plays a catalytic role in the decay of CH_2_OO.^46,47^ The reaction schematics, including the optimized geometries and the potential energy surface of CI2 with H_2_O, are presented in Fig. 7 and S5, respectively.

The O atom in H_2_O reacts with the C atom in the –CH group, and one of the H atoms in H_2_O shifts to the terminal-O atom in CI2 at the same time to generate intermediate D-IM1 through transition state D-TS1 crossing a free energy barrier of 13.07 kcal mol^−1^. The lengths of the new formations (C–O and O–H bonds) and the broken O–H bond are 1.960, 1.734 and 1.008 Å, respectively. D-IM1, with the internal energy of 50.00 kcal mol^−1^, could decompose to D-P1 (CF_3_COOH + H_2_O) through transition state D-TS2. This involves the H atom in the OH group shifting to the middle-O atom in the –COOH group, accompanied by the breaking of the C–O bond through transition state D-TS2, surmounting a free energy barrier of 47.03 kcal mol^−1^. In addition, D-IM1 can be reacted with monomolecular water *via* a six-membered ring transition state (D-TS3) to generate D-P1, and this is a two-hydrogen migration process. The free energy barrier of D-IM1 → D-TS3 → D-P1 is 31.93 kcal mol^−1^, which is 15.10 kcal mol^−1^ lower than that of D-IM1 → D-TS2 → D-P1, which indicates that the water molecule plays a catalytic role in this process. D-IM1 can also continue to react with the monomolecular water *via* a six-membered ring (D-TS4) to generate D-P2 (CF_3_CHO + H_2_O_2_). The free energy of D-IM1 → D-TS4 → D-P2 (CF_3_CHO + H_2_O_2_) is 46.58 kcal mol^−1^, which is 0.45 kcal mol^−1^ lower than that of D-IM1 → D-TS2 → D-P1 (CF_3_COOH + H_2_O). This is also a double hydrogen migration process, in which water plays a catalytic role. Alternatively, CI2 can directly react with the water dimer to form D-IM1 + H_2_O *via* transition state D-TS5. In the reaction of CI2 with the water dimer, a water molecule acts as a reactant with CI2, and the other water molecule performs proton exchange. The free energy barrier of this process is 5.69 kcal mol^−1^, which is 7.38 kcal mol^−1^ lower than that of CI2 + H_2_O → D-TS1 → D-IM1. Therefore, the reaction of CI2 with the water dimer is more feasible. Furthermore, it is shown that water autocatalysis will occur in CI with H_2_O reactions and that the atmospheric resources of H_2_O_2_ are realized through these reactions.

#### 3.4.5 Reaction mechanism of CI2 with CH_2_O

Previous investigations^48–50^ have shown that CH_2_OO with some aldehydes can prioritize generating a five-membered ring POZ, and then carry out further reactions. The reaction schematics, including the optimized geometries and the potential energy surface of CI2 with CH_2_O, are presented in Fig. 8 and S6, respectively. Two distinct reaction pathways (Path E1 and Path E2) have been discovered in the CI2 with CH_2_O reaction. The C and O atoms in CH_2_O can simultaneously be added to the terminal-O atom and C atoms in the –CH group in CI2 (Path E1) to generate intermediate E-IM1, respectively, or they can be added to the C atoms in the –CH group and terminal-O atoms in CI2 (Path E2) to generate intermediate IM1, respectively. The transition states corresponding to the above two processes are E-TS1 and TS3, respectively, with corresponding free energy barriers of 4.14 and 27.14 kcal mol^−1^, respectively. Therefore, the reaction pathway of Path E1 is preferred over the reaction pathway of Path E2. E-IM1 can simultaneously break O–O and C–O to generate E-P1 (CF_3_CHO + HCOOH) through E-TS2. The process of E-IM1 → E-TS2 → E-P1 needs to cross a free energy barrier of 49.11 kcal mol^−1^, and therefore, the subsequent reaction of E-IM1 makes a small contribution to the reaction. The subsequent reaction of IM1 has been discussed in Section 3.2.

#### 3.4.6 Reaction mechanism of CI2 with O_2_

CI2 reacts with high concentrations of O_2_, and the reaction schematics, including optimized geometries and PES, are presented in Fig. 9 and S7, respectively. As for Path F1, the cycloaddition intermediate (F-IM1) is directly generated with a barrier-free exothermic process, with a relative energy of 47.66 kcal mol^−1^. F-IM1, with the relative energy of −46.48 kcal mol^−1^, could simultaneously break the C–O and O–O bonds to generate F-P1 (CF_3_CHO + O_3_), easily surmounting a free energy barrier of 14.87 kcal mol^−1^. Path F2 involves the oxygen atoms in O_2_ attacking the terminal O atoms in CI2, kicking off CF_3_CHO to give out O_3_ radical through transition state F-TS2. The CI2 → F-TS2 → F-P1 (CF_3_CHO + O_3_) process requires overcoming the free energy barrier of 22.71 kcal mol^−1^. The generation of O_3_ results in the atmospheric cycling reaction of O_3_ with CF_3_CH=CH_2_. The results of Path F1 and Path F2 supply a resource for the generation of O_3_, which is crucial for understanding O_3_ pollution in specific regions. This result provides an important basis for formulating ozone pollution prevention and control measures and then taking corresponding emission reduction measures.

### 3.5 Kinetics

**Scheme 1:**
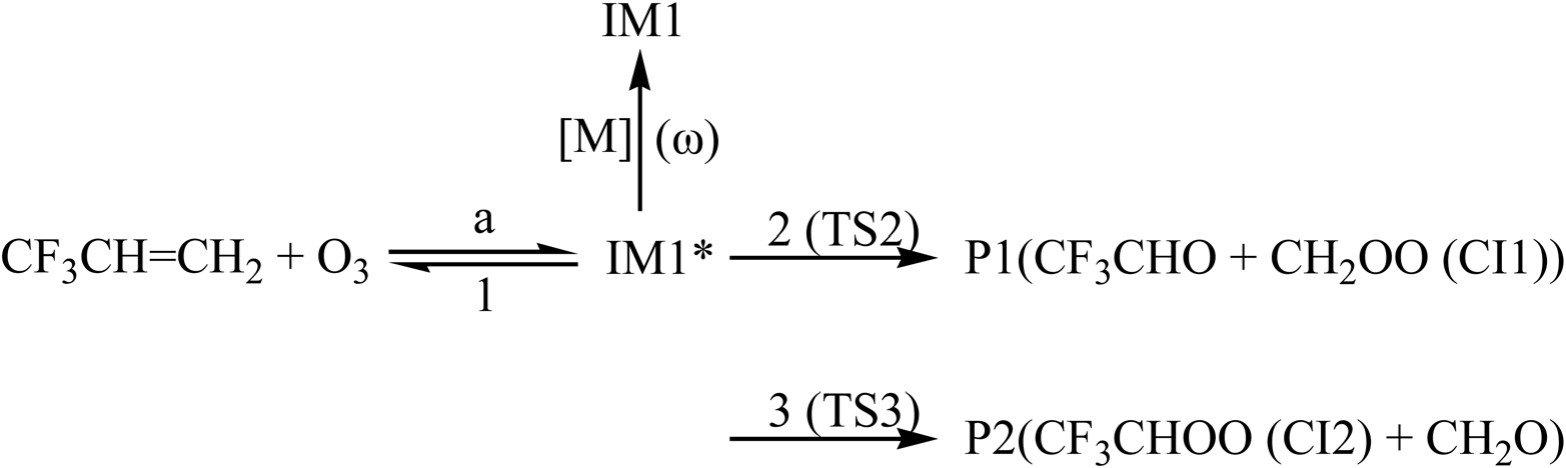


**Scheme 2:**
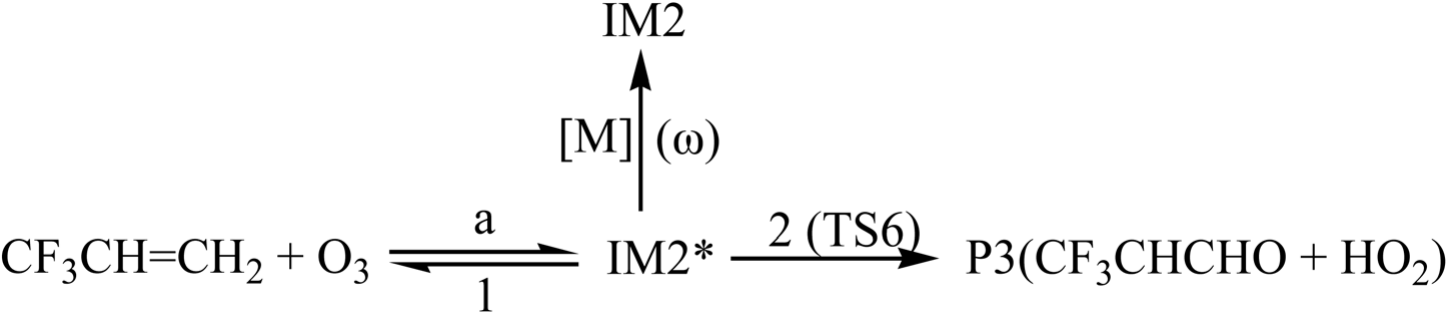


**Scheme 3:**
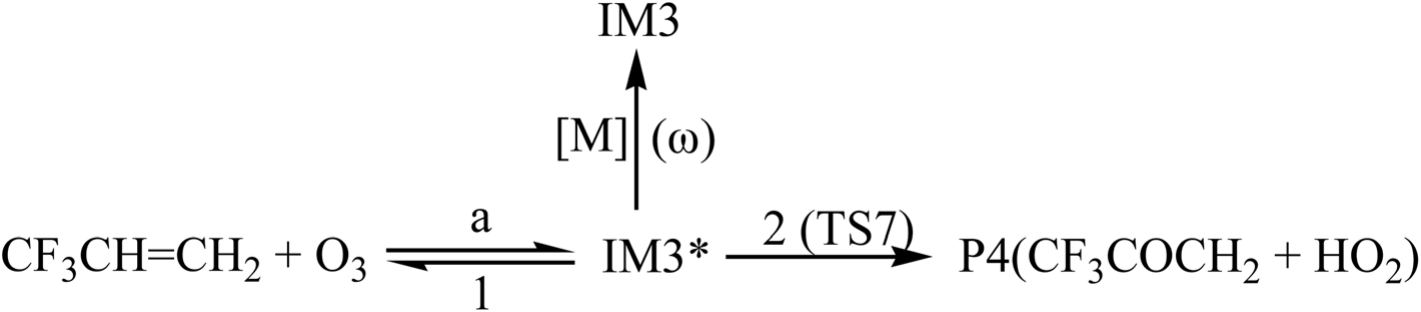


In order to understand reaction kinetics and compare the rate constants with experimental values, as well as guide the experimental investigation for the O_3_ + CF_3_CH=CH_2_ reaction, the temperature dependence of the total and individual pathways is computed at 200–3000 K using the RRKM theory for the primary pathways (Schemes 1–3), which are considered in the following computations:

Where * represents the vibrational excitation of the intermediate (IM1). Steady-state approximation for the energized intermediate (IM1*) leads to the following expressions:1
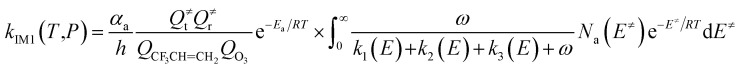
2
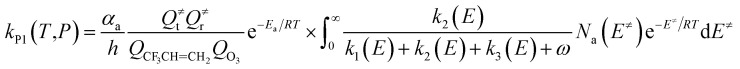
3
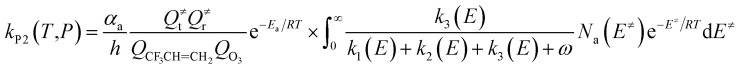


For Scheme 24
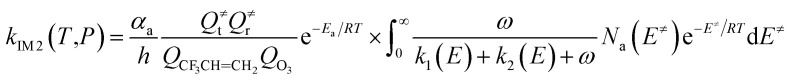
5
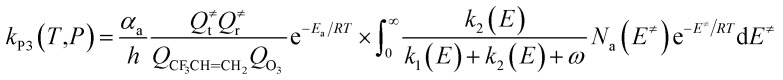


For Scheme 36
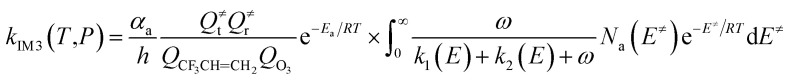
7
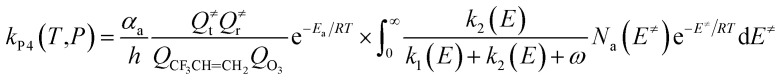


The microcanonical rate constant is calculated using the RRKM theory as follows:

8 *k*_*i*_(*E*) = *α*_*i*_*C*_*i*_*N*_*i*_(*E*^≠^_*i*_)/*hρ*_*j*_(*E*_*j*_)

In the above equations, *α*_a_ is the statistical factor for reaction Path a, and *α*_*i*_ is the statistical factor (degeneracy) for the *i*th reaction path; *E*_a_ is the energy barrier for reaction step a. *Q*_O_3__ and *Q*_CF_3_CH=CH_2__ are the total partition functions of O_3_ and CF_3_CH=CH_2_, respectively; *Q*^≠^_t_ and *Q*^≠^_r_ are the translational and rotational partition functions of the entrance transition states. *N*_a_(*E*^≠^) is the number of states for the association transition state with excess energy (*E*^≠^) above the association barrier. *k*_*i*_(*E*) is the energy-specific rate constant for the *i*th channel, and *C*_*i*_ is the ratio of the overall rotational partition function of TS_*i*_ and IM_*j*_; *N*_*i*_(*E*^≠^_*i*_) is the number of states at the energy above the barrier height for transition state *i*; *ρ*_*j*_(*E*_*j*_) is the density of states at energy *E*_*j*_ of the intermediate. The density of states and the number of states are calculated using the extended Beyer–Swinehart algorithm.

**Fig. 10:**
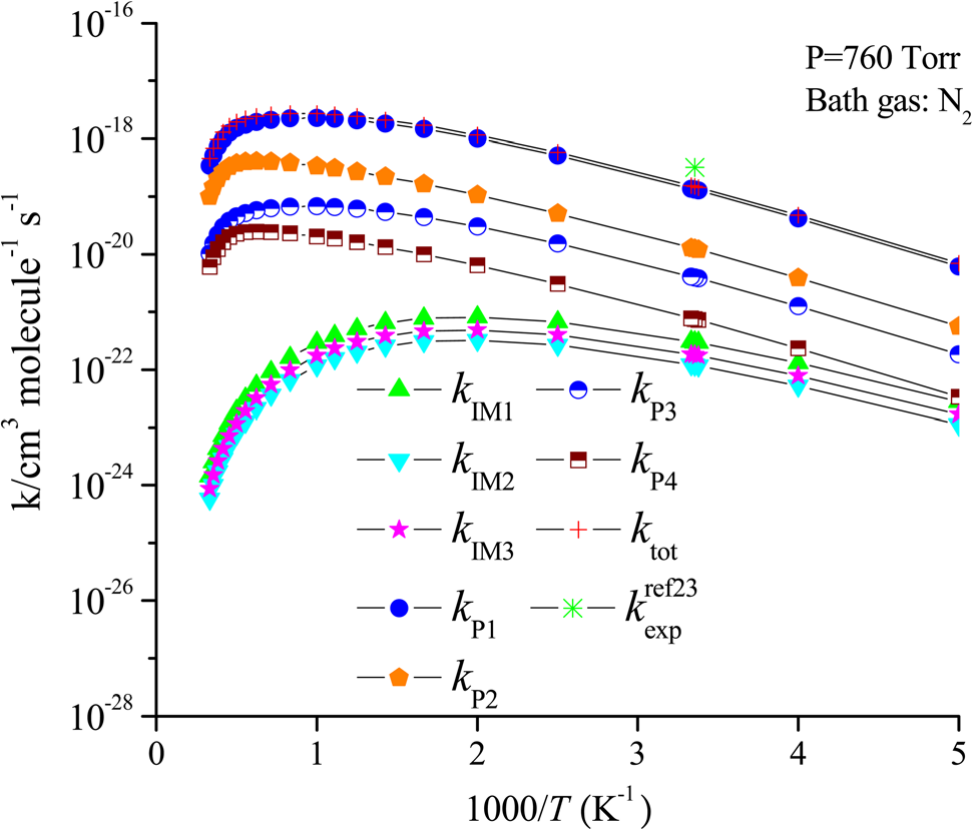
Temperature dependence of the total and individual rate constants for the trifluoropropene with ozone reaction at 760 torr N_2_.

**Fig. 11:**
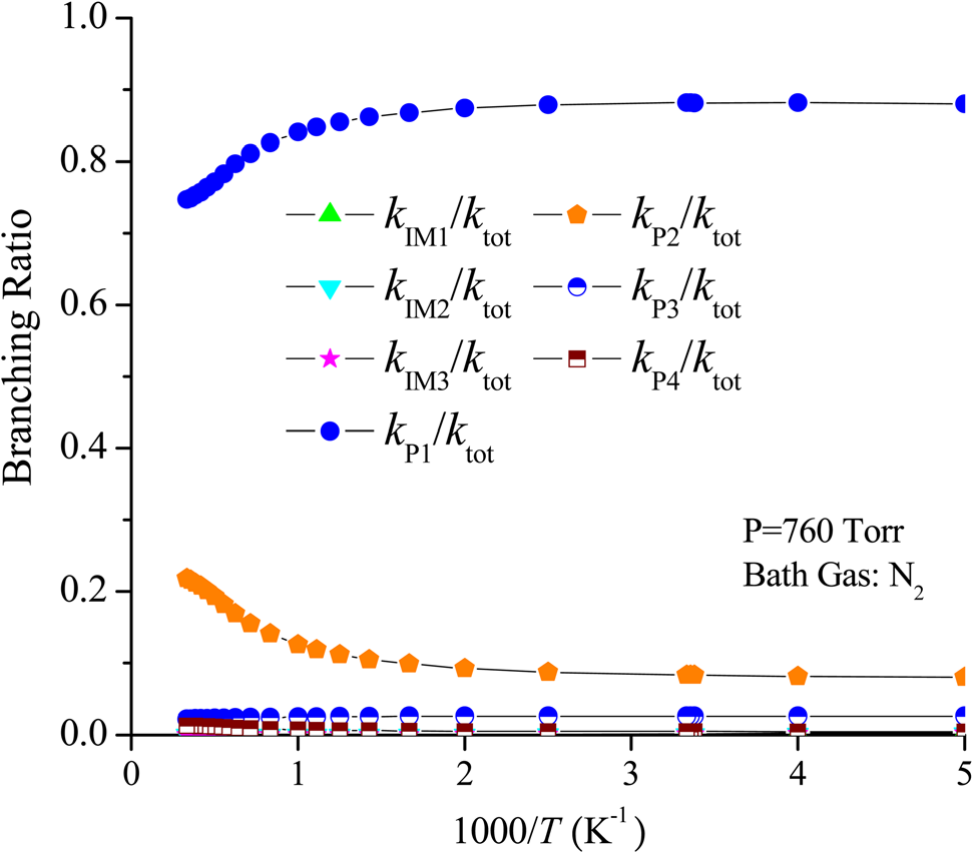
Branching ratios of the important product channels for the trifluoropropene with ozone reaction in the temperature range of 200–3000 K at 760 torr pressure of N_2_.

For the CF_3_CH=CH_2_ + O_3_ reaction, the rate constants of the generation of P1, P2, P3, P4, IM1, IM2 and IM3 are denoted as *k*_P1_, *k*_P2_, *k*_P3_, *k*_P4_, *k*_IM1_, *k*_IM2_ and *k*_IM3_, respectively, and the total rate constants are denoted as *k*_tot_, *k*_tot_ = *k*_P1_ + *k*_P2_ + *k*_P3_ + *k*_P4_ + *k*_IM1_ + *k*_IM2_ + *k*_IM3_, respectively. The temperature dependences of the total rate constants (*k*_tot_) and individual rate constants (*k*_P1_, *k*_P2_, *k*_P3_, *k*_P4_, *k*_IM1_, *k*_IM2_ and *k*_IM3_) at 760 torr are presented in Fig. 10. The computed *k*_tot_ at 298 K and 760 torr is 1.50 × 10^−19^ cm^3^ per molecule per s, which is overestimated almost two times at 298 K relative to the data reported by Sulbaek Andersen *et al.*^17^ in 2005 (*k*_tot_ = (3.50 ± 0.30) × 10^−19^ cm^3^ per molecule per s). The difference in the above result may be due to the bath gas, experimental conditions and measurement technique. The total rate constants and the rate constants for the generation of products P1, P2, P3 and P4, as well as collisional stabilization channels, increase at first but decrease rapidly with increasing temperatures. The branching ratios for the primary products are also displayed in Fig. 11. The primary product is P1 (CF_3_CHO + CH_2_OO (CI1)), and the pathway for generating P2 (CF_3_CHOO (CI2) + CH_2_O) contributes to the reaction to some extent over the entire temperature range. The contribution of the pathways for generating IM1, IM2, IM3, P3, and P4 to the reaction is negligible.

We also compute the rate constants for CI2 (CF_3_CHOO) with a variety of atmospheric species (NO, NO_2_, CH_2_O, H_2_O, SO_2_ and O_2_) at 298 K and 1 atm using the RRKM theory. The detailed computation process is similar to the title reaction. The rate constants for CI2 with NO, NO_2_, CH_2_O, H_2_O, SO_2_ and O_2_ are 5.21 × 10^−16^ cm^3^ per molecule per s, 1.22 × 10^−19^ cm^3^ per molecule per s, 7.25 × 10^−12^ cm^3^ per molecule per s, 3.14 × 10^−18^ cm^3^ per molecule per s, 3.03 × 10^−11^ cm^3^ per molecule per s, 3.11 × 10^−9^ cm^3^ per molecule per s, respectively. Therefore, the CI2 with O_2_ reaction is the fastest path, followed by those with SO_2_ and CH_2_O. Previous studies on the kinetics of the Criegee intermediate (CF_3_CHOO, CH_2_OO and (CH_3_)_2_COO) with a variety of atmospheric species (NO, NO_2_, CH_2_O, H_2_O, SO_2_ and O_2_)^51,52^ are provided in the SI (Table S2) and constitute valid evidence for assessing the transport and degradation of CF_3_CH=CH_2_. As shown in Table S2, because the CF_3_ radical with relatively high electronegativity strongly attracts electrons located at the C=C double bond, the electron density on the C=C double bond is reduced. This leads to a decrease in the reactivity of the C atom of the C=C double bond attack on the O atom of O_3_; however, the –(CH_3_)_2_ group is the donor species, which increases the rate constants. Thus, theoretically, the trend of the reaction rate constant is *k*((CH_3_)_2_COO) > *k*(CH_2_OO) > *k*(CF_3_CH=CH_2_), which is quantitatively in line with the experimental results.

The atmospheric lifetime of CF_3_CH=CH_2_ induced by O_3_ can be computed using the following formula: *τ* = (*k*_tot_[O_3_])^−1^, where *k*_tot_ is the total rate coefficient for CF_3_CH=CH_2_ with O_3_ at 298 K (1.50 × 10^−19^ cm^3^ per molecule per s), and [O_3_] is the atmospheric concentration of the ozone. The average concentration of O_3_ is 1.00 × 10^12^ molecule per cm^3^.^53^ At a normal temperature and pressure, the atmospheric lifetime of CF_3_CH=CH_2_ induced by O_3_ is estimated to be 77.16 days, which is consistent with the experimental data (70 days). It is observed that CF_3_CH=CH_2_ can be degraded to relatively small molecules in the atmosphere.

## 4. Conclusion

In this study, the ozonation reaction mechanism of trifluoropropene was investigated using quantum chemistry methods. Addition/elimination, H-extraction and substitution mechanisms were discovered in the reaction of CF_3_CH=CH_2_ with O_3_, and the addition/elimination mechanism was dominant. The computed rate constants and atmospheric lifetime at a normal temperature and pressure are 1.50 × 10^−19^ cm^3^ per molecule per s and 77.16 days, respectively, which are consistent with the experimental data ((3.50 ± 0.30) × 10^−19^ cm^3^ per molecule per s and 70 days).

We also studied the reaction mechanism between the Criegee intermediates and atmospheric substances (NO, NO_2_, CH_2_O, H_2_O, SO_2_ and O_2_) and summarized the reaction characteristics. The reactions between the Criegee intermediates and NO, NO_2_, and SO_2_ are some of the sources of secondary organic aerosols in the atmosphere. Water has a self-catalytic effect in the reaction between H_2_O and the Criegee intermediate; acid and H_2_O were discovered in the product. Secondary ozone oxides (SOZ) can also be formed by reacting the Criegee intermediates with CH_2_O. The addition reaction between O_2_ and the Criegee intermediate could re-release ozone, achieving the atmospheric circulation reaction of ozone. The computed results manifested that the reaction of CI2 with O_2_ is the fastest path, followed by those with SO_2_ and CH_2_O.

The present investigation supplies many mechanical and kinetic data that could ascertain the degradation channels of CF_3_CH=CH_2_ by ozonolysis. Some significant conclusions could be obtained from the available data. CF_3_CH=CH_2_ is degraded through the reaction with O_3_ in the troposphere to generate some products, which would result in the generation of secondary organic aerosols. These computations indicate the formation of Criegee intermediates, peroxynitrates, and peroxysulfates with environmental concerns.

## Author contributions

The authors declared that all the co-authors are aware of and approve of the submission.

## Conflicts of interest

The authors declare that they have no known competing financial interests or personal relationships that could have appeared to influence the work reported in this paper.

## Supplementary Material

RA-015-D5RA07265D-s001

## Data Availability

The data that support the findings of this study are available on request from the corresponding author. Supplementary information (SI) is available. See DOI: https://doi.org/10.1039/d5ra07265d.
