# Supplementary material for: Theoretical study on the generation of Criegee intermediates from the ozonolysis of trifluoropropene (CF3CH[double bond, length as m-dash]CH2)
Source: RSC Adv. 2025 Dec 15;15(58):50257–69. doi: 10.1039/d5ra07265d (PMC12705229; doi:10.1039/d5ra07265d)
Supplement: RA-015-D5RA07265D-s001 [file RA-015-D5RA07265D-s001.pdf]

Supplementary Information (SI) for RSC Advances.
This journal is © The Royal Society of Chemistry 2025

Theoretical study on the generation of Criegee intermediates from ozonolysis of Trifluoropropene ($CF_3CH{=}CH_2$)

Yunju Zhang,[1*] Meilian Zhao,[2] Cen Yao[3*], Zhiguo Wang,[1] Yuxi Sun,[1]

[1]*Key Laboratory of Photoinduced Functional Materials, Key Laboratory of Inorganic Materials Preparation and Synthesis, Mianyang Normal University, Mianyang 621000, PR China*

[2]*Shool of Public Health , Chengdu University of Traditional Chinese Medicine, ChengDu PR China*

[3]*School-enterprise Joint Technology Innovation Laboratory of Novel Molecular Functional Materials of Jilin Province, Institute of Chemical and Industrial Bioengineering, Jilin Engineering Normal University, Changchun 130052, China*

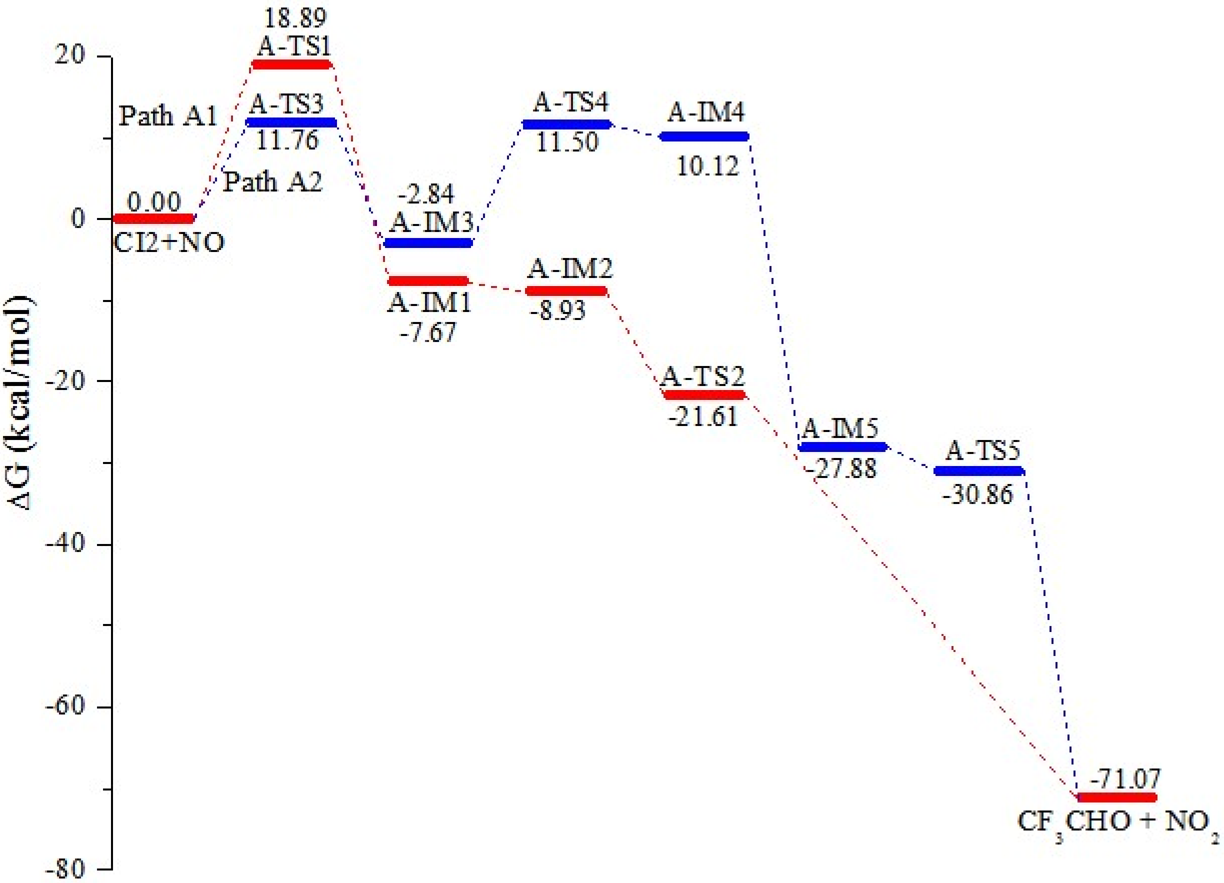

Figure S1: Potential energy surface for the reaction of CI2 (CF_3CHOO) with NO calculated at the CCSD(T)/6-311++g(d, p)//M06-2X/6-311++g(d, p) level of theory.

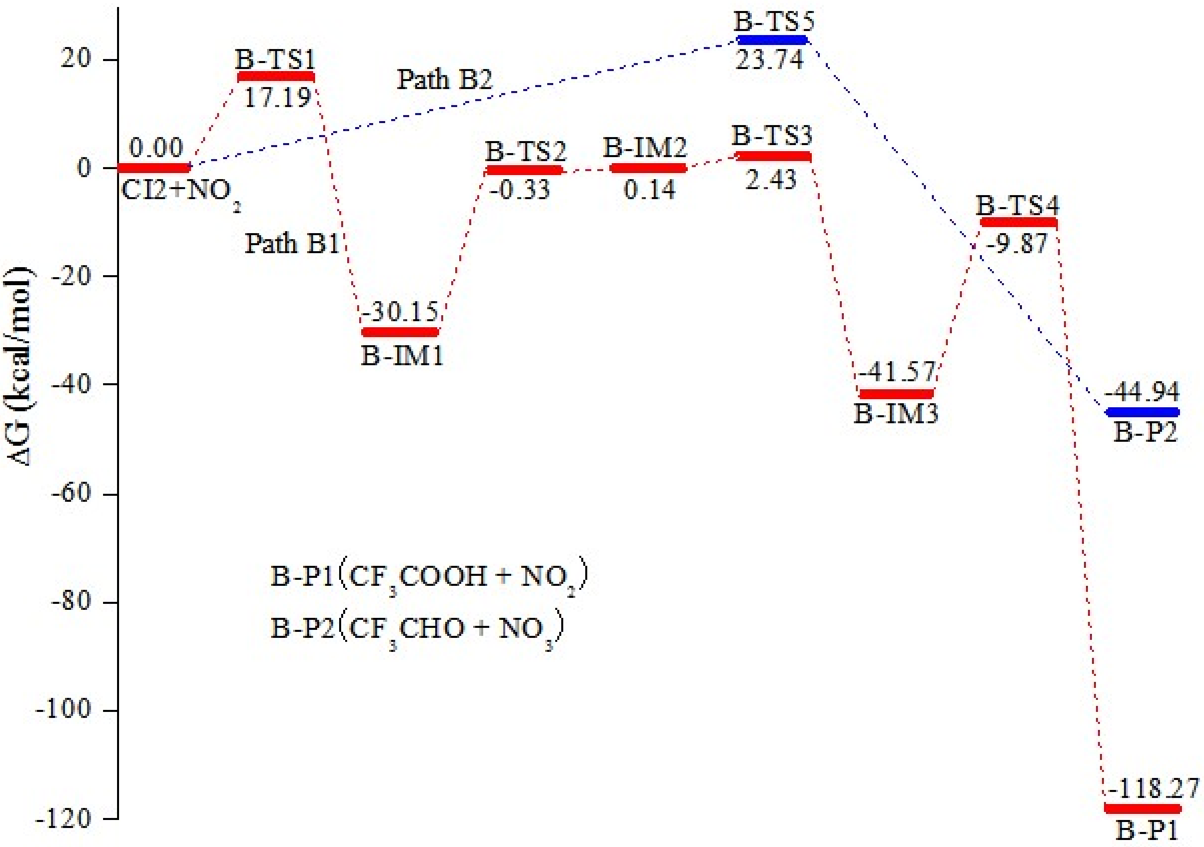

Figure S2: Potential energy surface for the reaction of CI2 (CF_3CHOO) with NO_2 calculated at the CCSD(T)/6-311++g(d, p)//M06-2X/6-311++g(d, p) level of theory.

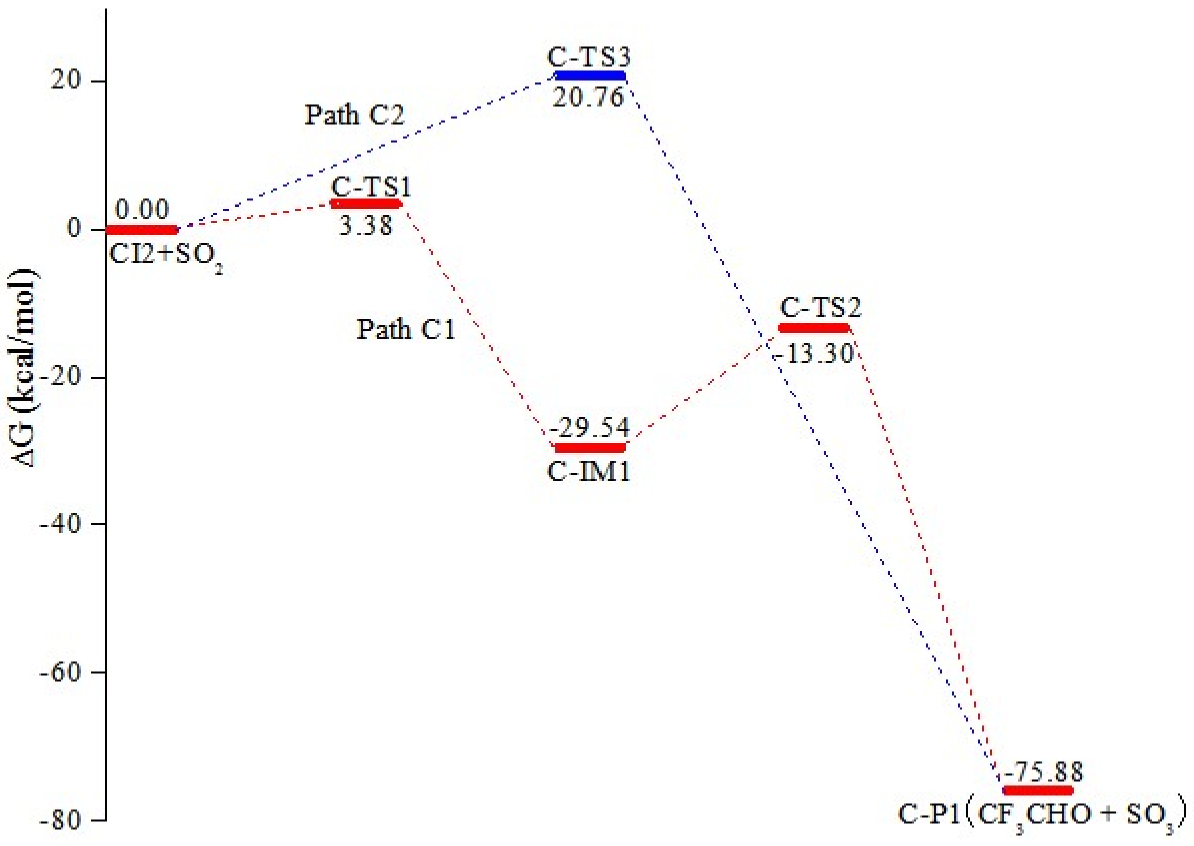

Figure S3: Potential energy surface for the reaction of CI2 (CF_3CHOO) with SO_2 calculated at the CCSD(T)/6-311++g(d, p)//M06-2X/6-311++g(d, p) level of theory.

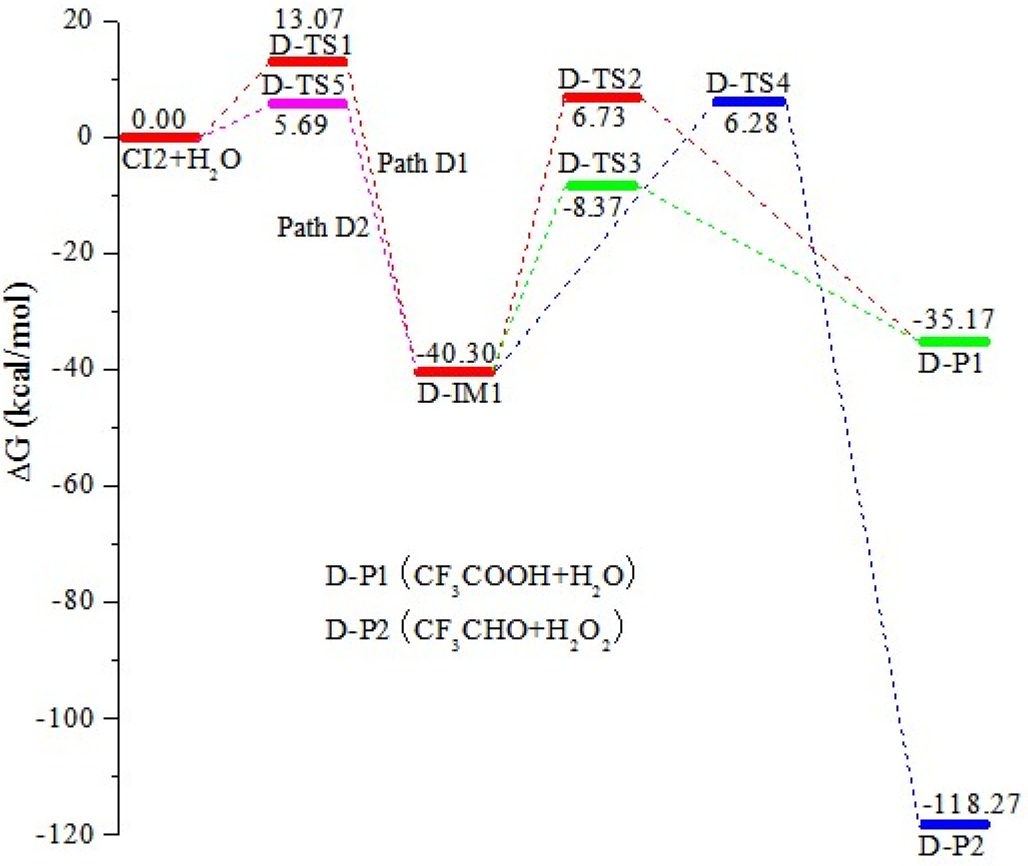

Figure S4: Potential energy surface for the reaction of CI2 (CF_3CHOO) with H_2O and $2H_2O$ calculated at the CCSD(T)/6-311++g(d, p)//M06-2X/6-311++g(d, p) level of theory.

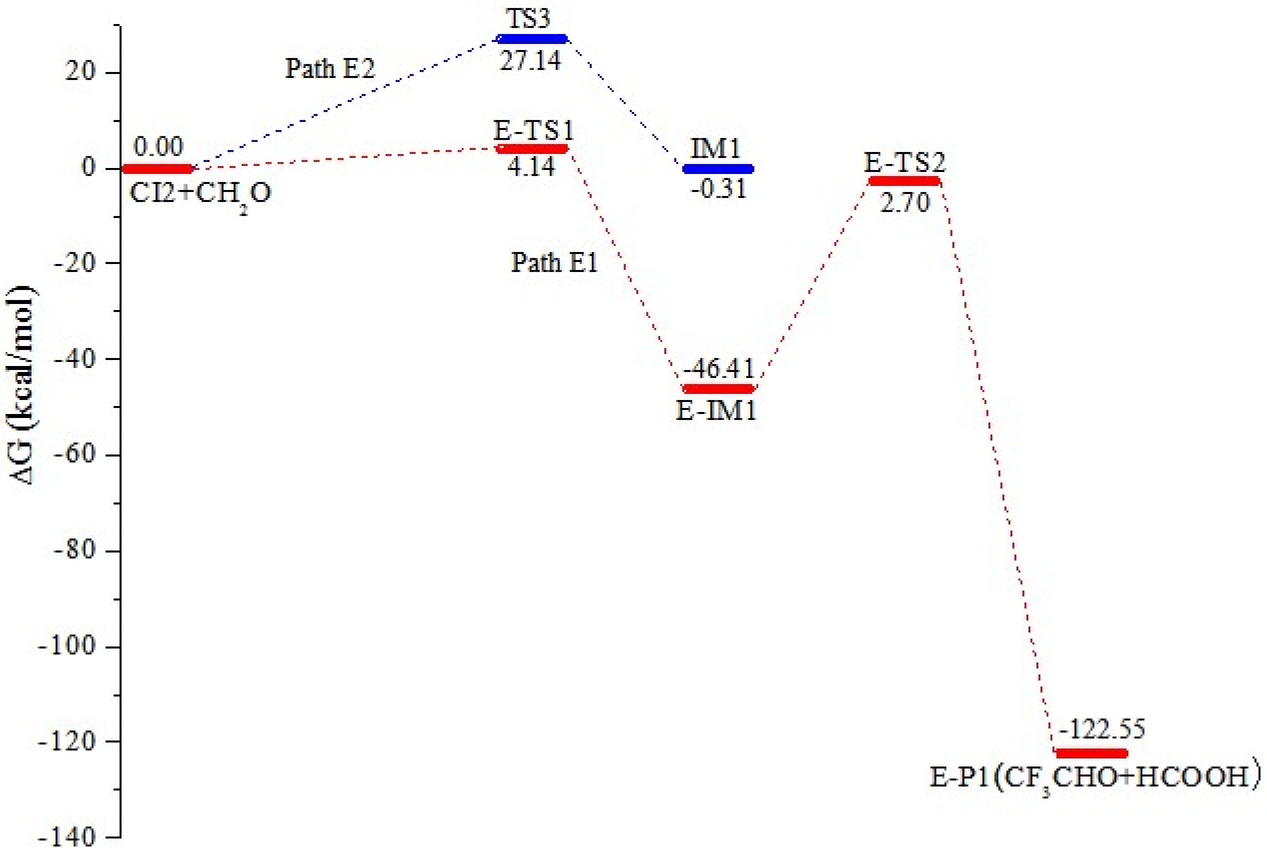

Figure S5: Potential energy surface for the reaction of CI2 (CF_3CHOO) with CH_2O calculated at the CCSD(T)/6-311++g(d, p)//M06-2X/6-311++g(d, p) level of theory.

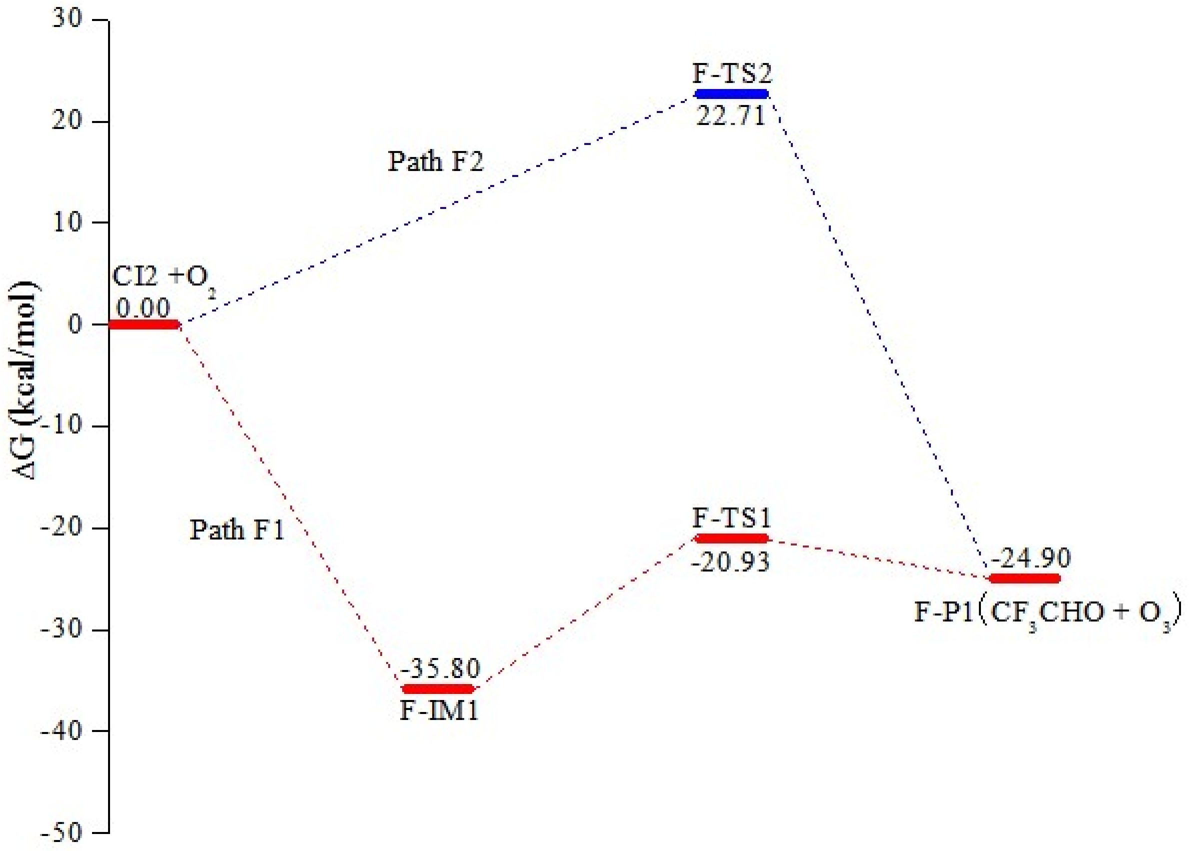

Figure S6: Potential energy surface for the reaction of CI2 (CF_3CHOO) with O_2 calculated at the CCSD(T)/6-311++g(d, p)//M06-2X/6-311++g(d, p) level of theory.

Table S1: Relative energies, reaction enthalpies and Gibbs free energy of all the species involving in the reactions of CI2 (CF_3CHOO) with NO, NO_2, SO_2, H_2O, CH_2O and O_2 at 298 K (in kcal/mol)

Species	ΔE	ΔH	ΔG
CI2 + NO	0.00	0.00	0.00
A-IM1	-18.30	-19.56	-7.67
A-IM2	-18.81	-19.61	-8.93
A-IM3	-12.75	-13.45	-2.84
A-IM4	-0.32	-1.46	10.12
A-IM5	-40.92	-41.87	-30.86
A-TS1	8.74	7.73	18.89
A-TS2	-31.94	-32.93	-21.61
A-TS3	2.94	2.69	11.76
A-TS4	1.06	-0.13	11.50
A-TS5	-40.92	-41.87	-30.86
A-P1(CF_3CHO + NO_2)	-69.69	-69.80	-71.07
CI2 + NO_2	0.00	0.00	0.00
B-IM1	-40.63	-40.94	-30.15
B-IM2	-11.81	-13.02	0.14
B-IM3	-52.65	-53.44	-41.57
B-TS1	6.53	6.10	17.19
B-TS2	-12.28	-13.46	-0.33

B-TS3	-9.54	-10.84	2.43
B-TS4	-20.65	-21.16	-9.87
B-TS5	14.60	14.79	23.74
B-P1: (CF_3COOH + NO_2)	-117.89	-117.88	-118.27
B-P2: (CF_3CHO + NO_3)	-44.20	-44.32	-44.94
CI2 + SO_2	0.00	0.00	0.00
C-IM1	-41.86	-42.85	-29.54
C-TS1	-9.23	-10.36	3.38
C-TS2	-25.63	-26.52	-13.30
C-TS3	10.58	10.63	20.76
C-P1: (CF_3CHO + SO_3)	-75.20	-75.36	-75.88
CI2 + H_2O	0.00	0.00	0.00
D-IM1	-50.00	-51.33	-40.30
D-TS1	3.13	1.58	13.07
D-TS2	-3.05	-4.40	6.73
D-TS3	-28.01	-30.94	-8.37
D-TS4	-13.06	-15.59	6.28
D-TS5	-13.52	-15.78	5.69
D-P1: (CF_3COOH + H_2O)	-33.00	-33.25	-35.17
D-P2: (CF_3CHO + H_2O_2)	-117.89	-117.88	-118.27
CI2 + CH_2O	0.00	0.00	0.00
E-IM1	-58.10	-59.71	-46.41

E-TS1	-6.46	-7.33	4.14
E-TS2	-14.33	-15.71	2.70
E-P1: (CF_3CHO + HCOOH	-121.34	-121.62	-122.55
CI2 + O_2	0.00	0.00	0.00
F-IM1	-46.28	-47.66	-35.80
F-TS1	-31.27	-32.52	-20.93
F-TS2	15.34	15.37	22.71
F-P1: (CF_3CHO + O_3)	-22.21	-22.28	-24.90
